# Co-Cultured Continuously Bioluminescent Human Cells as Bioreporters for the Detection of Prodrug Therapeutic Impact Pre- and Post-Metabolism

**DOI:** 10.3390/s17122827

**Published:** 2017-12-06

**Authors:** Tingting Xu, Michael Conway, Ashley Frank, Steven Ripp, Gary Sayler, Dan Close

**Affiliations:** 1 Center for Environmental Biotechnology, The University of Tennessee, Knoxville, TN 37996, USA; txu2@utk.edu (T.X.); saripp@utk.edu (S.R.); 2 490 BioTech, Inc., Knoxville, TN 37996, USA; michael.conway@490biotech.com (M.C.); gary.sayler@490biotech.com (G.S.); 3 Entomology Department, Cornell University, Ithaca, NY 14850, USA; amf336@cornell.edu

**Keywords:** bioluminescence, bacterial luciferase, pharmaceutical development, optical imaging

## Abstract

Modern drug discovery workflows require assay systems capable of replicating the complex interactions of multiple tissue types, but that can still function under high throughput conditions. In this work, we evaluate the use of substrate-free autobioluminescence in human cell lines to support the performance of these assays with reduced economical and logistical restrictions relative to substrate-requiring bioluminescent reporter systems. The use of autobioluminescence was found to support assay functionality similar to existing luciferase reporter targets. The autobioluminescent assay format was observed to correlate strongly with general metabolic activity markers such as ATP content and the presence of reactive oxygen species, but not with secondary markers such as glutathione depletion. At the transcriptional level, autobioluminescent dynamics were most closely associated with expression of the CYP1A1 phase I detoxification pathway. These results suggest constitutively autobioluminescent cells can function as general metabolic activity bioreporters, while pairing expression of the autobioluminescent phenotype to detoxification pathway specific promoters could create more specific sensor systems.

## 1. Introduction

Drug discovery relies on a combination of in vitro cell culture-based and in vivo whole animal-based models to identify, validate, and ensure the safety of promising therapeutic agents. The upstream, in vitro testing component of this system serves as a high throughput triage, with an average of 10,000 molecules screened for each new lead compound developed [1]. As drug discovery becomes more complex, it is paramount that new in vitro assays systems be developed that can perform this screening at lower costs, with higher throughput, and with improved predictive abilities in order to achieve the economics necessary for the process to remain profitable. A significant stumbling block towards this end has been the lack of complexity and parallel systems interaction provided by these tests relative to their downstream, in vitro counterparts. 

This presents a challenge whereby increasing the complexity of the system adds cost and decreases throughput, and decreasing the complexity lowers its predictive ability and allows false positive compounds to proceed to the more expensive in vivo testing stage. This conundrum requires that complex systems be endowed with low-cost, high throughput technologies in order to provide both the scientific and economic power required to identify promising lead compounds and quickly move them into further testing. Furthermore, these systems must be human-relevant, since non-human, species-specific effects are responsible for up to 92% of failures at the clinical level [2,3,4].

One promising solution to this problem is the co-culture and simultaneous treatment of multiple tissue types to ascertain both the treatment effects of a compound on the individual tissues and the effects that the resultant, biotransformed metabolic breakdown products of each cell type have on their partner tissues. These systems, when combined with fluorescent and bioluminescent reporter genes for the detection of specific effects and general cellular health monitoring, provide a suitable tradeoff between complexity, human-relevance, and cost [5]. In this work, we evaluate the use of a human-optimized synthetic luciferase construct to increase the throughput and data output of these co-culture-based assays. This synthetic luciferase endows host cells with an autobioluminescent phenotype that continuously produces a bioluminescent signal representative of their real-time metabolic activity level without requiring any external stimulation [6]. 

The autonomous light production of this system is made possible by the co-expression of six individual genes from a single mRNA that is encoded as a human cell expression-optimized gene cassette [7]. Two of the six genes (*luxAB*) encode subunits for a dimeric luciferase. Three additional genes (*luxCDE*) encode for a multimeric substrate synthesis complex [8]. This complex produces a long chain aldehyde luciferin substrate from endogenous metabolites that the luciferase uses to produce the bioluminescent signal. The sixth gene (*frp*) encodes an oxidoreductase that recycles Flavin mononucleotide (FMN) generated in the bioluminescent reaction to the required cofactor, reduced Flavin mononucleotide (FMNH_2_) [9]. By encoding both the luciferase and the luciferin generation pathway, the synthetic luciferase cassette continuously produces an autobioluminescent signal that is modulated in real-time in response to changes in metabolic activity as dictated by corresponding changes of co-factor availability. This is the same strategy used in traditional firefly luciferase systems, where ATP is leveraged as a limiting reagent for light production to report the cell’s metabolic activity level. The continuous light production of the synthetic luciferase system has the potential to increase the amount of information that can be obtained at a comparable cost, and over a comparable timeframe, to existing screening regimens, while maintaining the critical advantage of presenting human species-specific data.

## 2. Materials and Methods 

### 2.1. Development of Autobioluminescent Cell Lines

Human breast cancer T47D and liver cancer HepG2 cells were obtained from the American Type Culture Collection. To develop cells with autobioluminescent phenotypes, they were transfected with a synthetic luciferase cassette (490 BioTech, Knoxville, TN, USA) using the Neon Transfection System (Thermo Scientific, Hampton, NH, USA). Immediately following electroporation, cells were plated in 10-cm tissue culture dishes containing fresh medium. To select for stable clones, electroporated cells were treated with Geneticin (G418, 500–750 µg/mL) for roughly two weeks until individual G418-resistant clones were formed. The clones were then expanded into individual lines and ranked for autobioluminescence. To evaluate light production, each clone was seeded at ~1 × 10^4^ cells/well in triplicate wells of a flat-bottom black 96-well plate and bioluminescence was measured in an IVIS Lumina (PerkinElmer, Waltham, MA, USA). The clone of each cell type displaying the greatest signal output was selected for the assays described below. 

### 2.2. ATP Content Screening

Autobioluminescent T47D or HepG2 cells were seeded at ~1 × 10^4^ or 2.5 × 10^4^ cells/well, respectively, in flat-bottom black 96-well plates and incubated under standard conditions (37 °C, 5% CO_2_). After overnight incubation, medium was removed from the cells and replaced with fresh medium containing Cytarabine, Methotrexate, or Oligomycin B (Sigma-Aldrich, Saint Louis, MO, USA) at concentrations ranging from 1 nM to 10 µM or with 0.1% Dimethyl sulfoxide (DMSO) as a control. Test compounds were prepared in DMSO and the final concentration of DMSO was 0.1% in all wells. Each compound was tested in triplicate plates and each concentration was tested in triplicate wells per plate. After dosing, cells were incubated under standard conditions for 24 h before they were interrogated in a Synergy2 plate reader (Bio-Tek, Winooski, VT, USA) using the CellTiter-Glo assay (Promega, Madison, WI, USA) according to the manufacturer’s protocol. 

### 2.3. Reactive Oxygen Species Screening

The fluorescent dichlorofluorescin diacetate (DCFH-DA) assay (abcam, Cambridge, UK) was used according to the manufacture’s 24–48 h treatment protocol to analyze the production of reactive oxygen species in cells exposed to Doxorubicin or tert-butyl H_2_O_2_ (TBHP). Autobioluminescent T47D or HepG2 cells were seeded at ~2.5 × 10^4^ cells/well in flat-bottom black 96-well plates and incubated under standard conditions. After overnight incubation and prior to compound treatment, attached cells were washed with 100 μL 1 × phosphate buffered saline (PBS) and then treated with 100 μL of the test compound at concentrations ranging from 1 nM to 10 µM, or with 0.1% DMSO as a control. Each compound was tested in triplicate plates and each concentration was tested in triplicate wells per plate. After treatment for 23 h, 100 μL of 60 μM 2′,7′-dichlorofluorescin diacetate (DCFH-DA) was added to each well and the cells were incubated for an additional 30–60 min. After 24 h of total incubation, fluorescence was measured using a 485 nm excitation and a 535 nm emission wavelength in the CLARIOstar plate reader (BMG Labtech Ortenberg, Germany). 

### 2.4. Glutathione Depletion Screening

The luminescent GSH-Glo assay (Promega) was used to analyze glutathione depletion in cells exposed to test compounds. Autobioluminescent T47D or HepG2 cells were seeded at ~1 × 10^4^ or 2.5 × 10^4^ cells/well, respectively, in flat-bottom black 96-well plates and incubated under standard conditions. After overnight incubation, the medium was removed from the cells and replaced with fresh medium containing Digoxin or Rotenone (Sigma-Aldrich) at concentrations ranging from 1 nM to 10 µM or with 0.1% DMSO as a control. Test compounds were prepared in DMSO and the final concentration of DMSO was 0.1% in wells treated with the test compounds. Each compound was tested in triplicate plates and each concentration was tested in triplicate wells per plate. After dosing, cells were incubated under standard conditions for 24 h before they were interrogated in a Synergy2 plate reader using the GSH-Glo according to the manufacturer’s protocol.

### 2.5. Detoxification Pathway Biomarker Activation Screening

Autobioluminescent T47D or HepG2 cells were seeded in triplicate into white 96-well plates at ~2.5 × 10^4^ cells/well and incubated under standard conditions. After overnight incubation, cells were dosed with serial dilutions of either β-naphthoflavone (b-NF), 3-methylcholanthrene (3-mc), Indirubin, or 2,3,7,8-tetrachlorodibenzo-p-dioxin (TCDD) (Sigma-Aldrich) at concentrations ranging from 1 nM to 10 µM, or with 0.1% DMSO as a control. The treated plates were immediately transferred to the CLARIOstar plate reader and assayed for autobioluminescent production using a 60 s/well integration every 60 min over a 24 h period. At the completion of the assay, significant changes in autobioluminescent output (defined as Student’s *t*-tests displaying a decrease in luminescent flux at *p* ≤ 0.05 as compared to cells treated with 0.1% DMSO) were noted. The assay was then repeated and, at the points where significant changes in autobioluminescent output were detected, RNA was extracted from the cells of that treatment group and its associated untreated control using a Cells-to-Ct kit (Ambion, Waltham, MA, USA). The recovered RNA was subjected to quantitative reverse transcription polymerase chain reaction (qRT-PCR) and transcript levels were assessed for the CYP1A1, CYP1A2, CYP2B6, and CYP3A4 phase I and SULT1A, SULT1A2, SULT1E1, SULT2A1, mGST1, GSTµ1, NAT1, and EPHX1 phase II detoxification enzymes. For all enzymes, previously validated qRT-PCR primers [10,11] were used to ensure faithful and exclusive amplification, and assessment of β-actin transcript levels was included for normalization of results. The expression level of each detoxification enzyme was normalized to β-actin expression using the ∆Cq method. These expression levels were then compared between samples (i.e., fold change in treated cells relative to untreated controls) using the ∆∆Cq analysis method.

### 2.6. Autobioluminescent Metabolic Activity Screening

To prepare cells for autobioluminescent metabolic activity screening assays, autobioluminescent T47D or HepG2 cells were seeded at ~1 × 10^4^ cells/well in flat-bottom black 96-well plates and incubated under standard conditions. After overnight incubation, cells were treated with test compounds at final concentrations ranging from 1 nM to 10 µM or with 0.1% DMSO as a control. Each compound was tested in triplicate plates and each concentration was tested in triplicate wells per plate. The treated plates were immediately transferred to the IVIS Lumina and assayed for autobioluminescent production every 15 min over a 24 h period. 

To detect post-biotransformed compound effects, the upstream cell type was first treated with the test compound as described above. After 24 h of treatment, the medium from the upstream cell type was transferred to the downstream cells. The treated downstream cells were immediately transferred to the IVIS Lumina and assayed for autobioluminescent production as described above.

### 2.7. Statistical Analysis

Test compound concentrations were assayed in triplicate. Means and standard deviations were calculated for all readings using these technical replicates. Student’s *t*-test was applied to compare treatment levels against vehicle controls using a significant *p* value ≤ 0.05. All errors were reported as the standard error of the mean (SEM), unless otherwise specified. Detoxification enzyme induction was identified by applying Student’s *t*-test to compare β-actin-normalized gene expression levels (ΔCq values) between treated and untreated cells. Significant induction was determined as those samples displaying a *p* value ≤ 0.05 and a ≥ 3-fold induction. Significant differences between the fold changes of different detoxification enzymes were calculated by applying Student’s *t*-test to their relative gene expression levels (ΔΔCq values) using *p* ≤ 0.05 as a significance cutoff.

## 3. Results

### 3.1. Development of Autobioluminescent Cell Lines

Following two weeks of G418 treatment, approximately 20 resistant clones were randomly selected from both the synthetic luciferase-transfected T47D and HepG2 cell lines and evaluated for bioluminescent production. For each cell line, the stable clone displaying the highest level of light production was chosen for the assays described in this manuscript. 

### 3.2. Detection of Pre- and Post-Biotransformed Compound Effects Using Autobioluminescent Cell Lines

**Figure 1:**
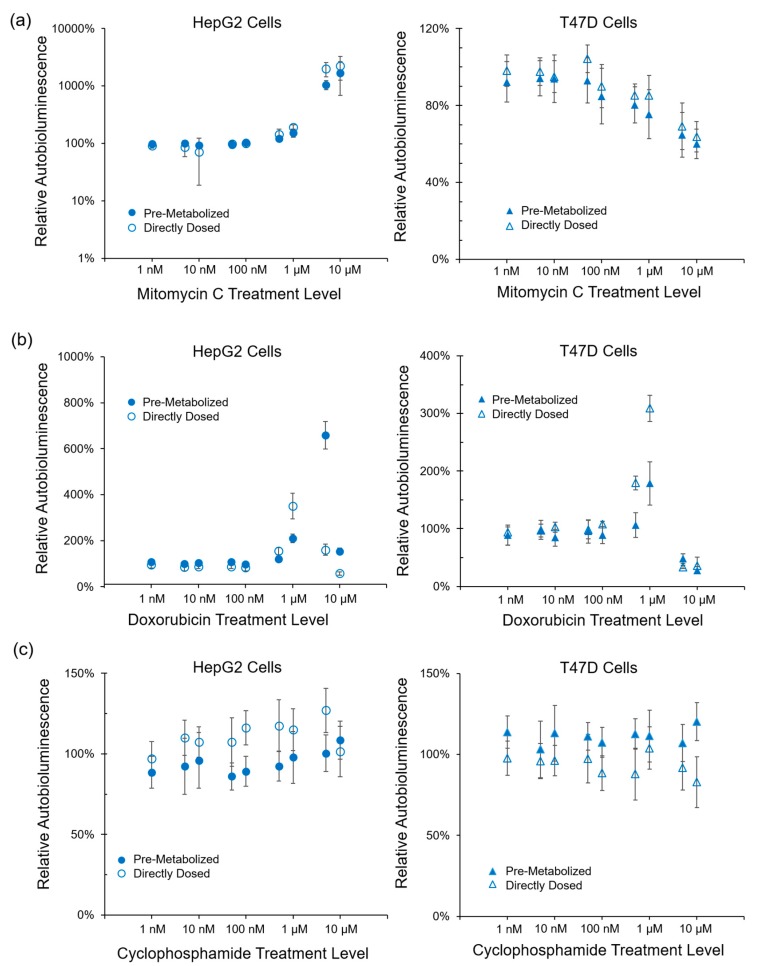
Autobioluminescent cells successfully detected both pre- and post-metabolized prodrug metabolic impacts from (**a**) the known cytotoxic compounds Mitomycin C and (**b**) Doxorubicin hydrochloride. (**c**) Treatment with Cyclophosphamide, which is known to become metabolically active only after liver cell metabolism, showed metabolic effects in the downstream human breast cancer (T47D) cell line, but not the upstream liver cancer (HepG2) cell line. Values are the averages of triplicate tests, with error represented as the standard error of the mean (SEM).

Treatment with the known cytotoxic compound Mitomycin C-induced metabolic impacts regardless of if it was provided directly to either of the autobioluminescent cell lines, or if it was first metabolized by one line, then exposed to the other (Figure 1a). Mitomycin C imparted a significant metabolic impact in HepG2 cells at treatment levels > 500 nM, regardless of if the compound was dosed directly, or pre-metabolized by the T47D cell line. T47D metabolism was significantly impacted by treatment levels ≥ 1 μM, with no difference if the compound was dosed directly or pre-metabolized by the HepG2 cell line. Doxorubicin hydrochloride treatment imparted a significant metabolic impact on both the HepG2 and T47D cell lines regardless of if it was dosed directly or pre-metabolized by the alternative cell type (Figure 1b). Pre-metabolism by the T47D cell line caused HepG2 metabolism to increase relative to the effect of directly dosing the cells, while the opposite was observed for T47D cells treated with Doxorubicin hydrochloride pre-metabolized by HepG2 cells. Treatment of the HepG2 liver cell line with Cyclophosphamide, which is known to become metabolically active only after liver cell metabolism [12], did not show any metabolic impact, regardless of if the compound was dosed directly or pre-metabolized by the T47D cell line. In contrast, pre-metabolism by the HepG2 cell line led to a metabolic impact on the T47D cell line that was not observed following direct dosing (Figure 1c).

### 3.3. Identification of the Mechanisms of Action Underlying Autobioluminescent Dynamics

**Figure 2:**
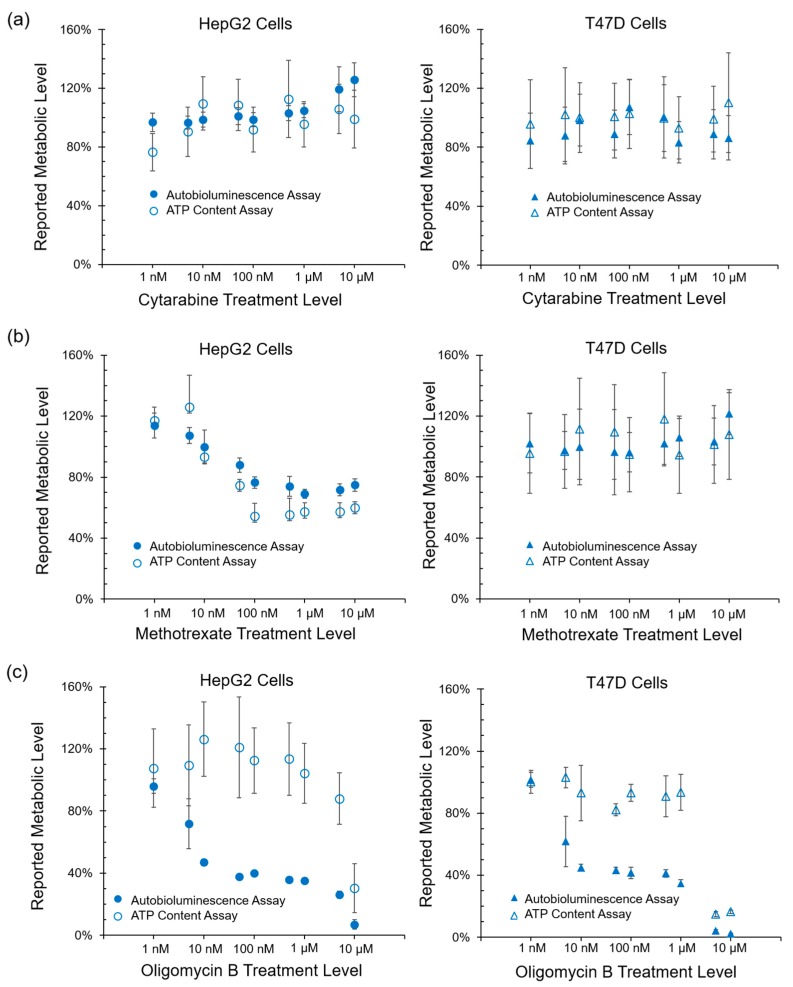
Detection of metabolic impact using autobioluminescence correlated with ATP content for both (**a**) Cytarabine and (**b**) Methotrexate, and was more sensitive for the detection of (**c**) Oligomycin B. Values are the averages of triplicate tests, with error represented as SEM.

For all tested compounds, autobioluminescent assays correlated strongly with ATP content assays. Cytarabine treatment, which does not decrease cellular ATP content [13], was not identified as decreasing metabolic activity using either ATP content or autobioluminescence for detection (Figure 2a). Treatment with Methotrexate, which alters the ATP content levels of HepG2 cells, but not T47D cells [14], resulted in similar responses between the two assays for each cell type (Figure 2b). Between the two approaches, autobioluminescence-based testing was found to be more sensitive than ATP depletion, as measured by the identification of metabolic impacts at lower compound doses than was observed using ATP content assays for detection of the same compound. This effect was best demonstrated by the detection of Oligomycin B in Figure 2c.

**Figure 3:**
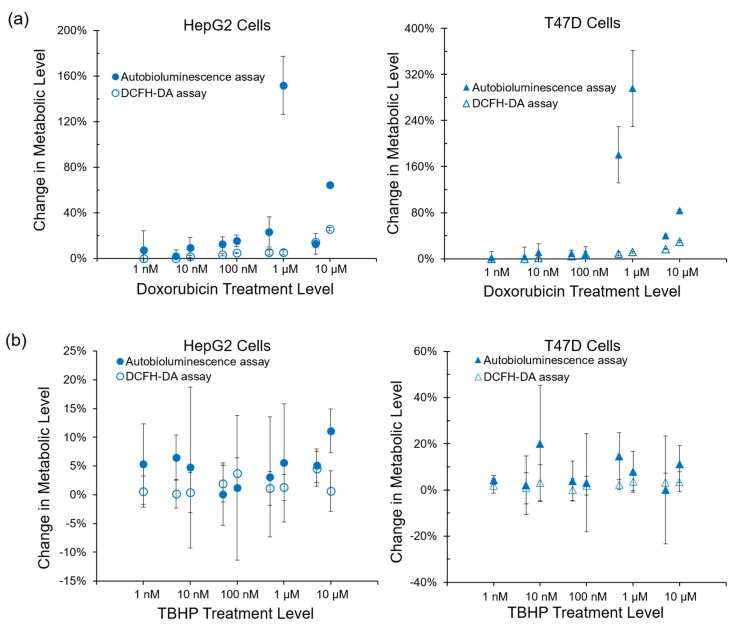
The autobioluminescence assay correlated with the fluorescent dichlorofluorescin diacetate (DCFH-DA) assay for the detection of reactive oxygen species as mediators of metabolic impact for both (**a**) Doxorubicin and (**b**) tert-butyl H_2_O_2_ (TBHP). Values are the averages of triplicate tests, with error represented as SEM.

**Figure 4:**
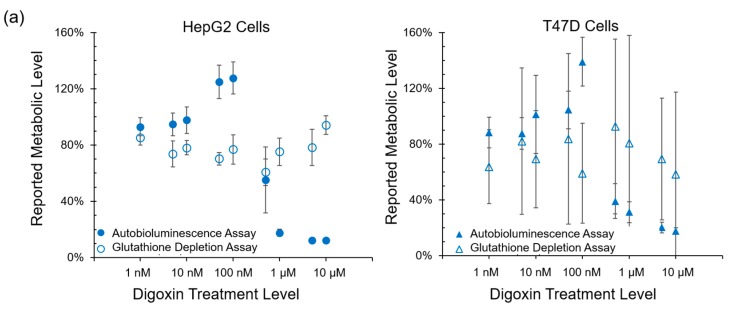

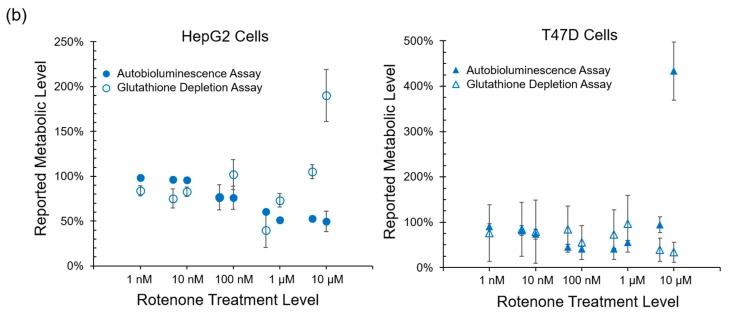
No correlation was observed between the results of autobioluminescent assays and glutathione depletion assays for following treatment with (**a**) Digoxin or (**b**) Rotenone. Values are the averages of triplicate tests, with error represented as SEM.

To determine if autobioluminescent response dynamics are correlated with the presence of reactive oxygen species, the autobioluminescent assay was compared with the fluorescent DCFH-DA assay, which is capable of directly measuring the level of a broad range of reactive species with diverse biological reactivities [15,16]. The autobioluminescent assay format detected metabolic effects from the same compounds as did the DCFH-DA assay, with the exception of TBHP treatment of the HepG2 cell line (Figure 3). In contrast to the strong correlations between autobioluminescence and ATP content or reactive oxidative species, no correlation was observed between the results of autobioluminescent assays and glutathione depletion assays following treatment with either Digoxin (Figure 4a) or Rotenone (Figure 4b).

### 3.4. Correlation between Autobioluminescet Dynamics Detoxification Pathway Activation

**Figure 5:**
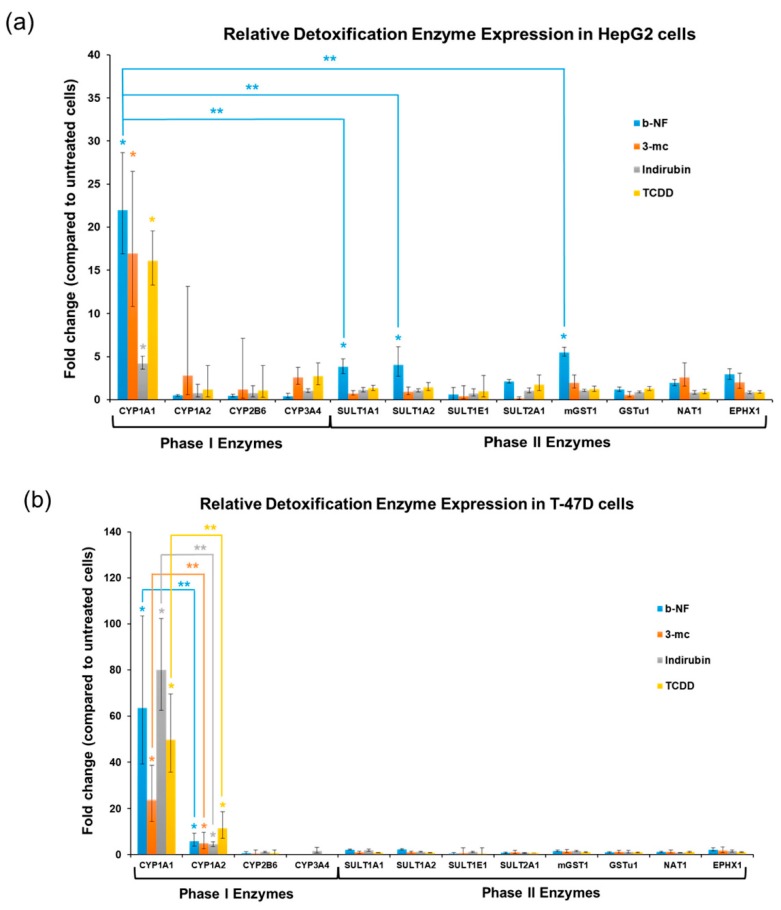
CYP1A1 was the most upregulated enzyme for both the (**a**) HepG2 and (**b**) T47D cell lines at the time autobioluminescent metabolic impact was reported. No significant upregulation of any Phase II detoxification enzyme was observed in the (**b**) T47D cell line, while SULT1A1, SULT1A2, and mGST1 were upregulated in the (**a**) HepG2 cell line. For each detoxification enzyme, significant induction in treated cells relative to their untreated controls (*p* ≤ 0.05) is denoted by a single asterisk (*). Under a given compound treatment condition, significant differences in the level of upregulation between different detoxification enzymes is indicated by a double-asterisk (**).

Autobioluminescent cells were treated with b-NF, 3-mc, Indirubin, or TCDD, and qRT-PCR was performed at the onset of autobioluminescent perturbation (defined as the earliest time point when autobioluminescent signal from treated cells was significantly different (*p* ≤ 0.05) from that of the untreated control cells) to correlate these changes with common phase I and phase II detoxification pathway biomarkers. The most upregulated enzyme in both the HepG2 (Figure 5a) and T47D (Figure 5b) cell lines at the time of autobioluminescent metabolic impact signaling was CYP1A1. This marker was significantly upregulated by all tested compounds in both cell lines. In the HepG2 cell line, CYP1A1 was the only phase I enzyme to demonstrate a ≥ 3-fold increase relative to its basal expression level in untreated control cells. In the T47D cell line, CYP1A2 was also observed to be significantly upregulated relative to untreated controls, however, the average expression of CYP1A1 was 48 (±12) times as great as that of CYP1A2 at the time of autobioluminescent signaling across all surveyed compounds. Neither the CYP2B6 nor CYP3A4 enzymes showed a significant increase in expression at the autobioluminescence-defined time of metabolic impact for either cell type under any of the treatment conditions. The only condition observed to trigger an increase in the transcription of phase II detoxification enzymes was treatment of the HepG2 cell line with 10 μM b-NF (Figure 5a). Under this test condition, the autobioluminescent response was found to correspond to the activation of SULT1A1, SULT1A2, and mGST1. Each of these enzymes was observed to be significantly induced under these conditions, however, none was more significantly activated than the other two. The relative increase in expression of these enzymes was lower than that of the CYP1A1 phase I enzyme, averaging only 4.5 (±0.5) fold over their untreated control states.

## 4. Discussion

The use of autobioluminescence as a metabolic activity reporter system has been used in a variety of cell types and applications [7,17,18,19,20,21,22], but has not been well-investigated for co-culture-based systems such as those used for drug discovery. In this work, we evaluated if this system could be used in this application, and if doing so could provide economical or logistical advantages relative to existing systems. The pre- and post-metabolism prodrug testing results (Figure 1b) suggest that this format can be used to determine both the immediate metabolic impact of a chemical and how dynamic this impact is when its metabolized products are exposed to other cell types. This ability to gather time-resolved pre- and post-metabolism data from a single assay, using only a single preparation of cells in one multi-well plate, is unique to the autobioluminescent assay format and provides advantages in cost, setup, and data output levels relative to alternative assay formats.

The autobioluminescent assay demonstrated the anticipated results following treatment with cytotoxic compounds, as demonstrated by reductions in metabolic activity from both cell lines following Mitomycin C and Doxorubicin hydrochloride treatment. Cyclophosphamide treatment was only observed to affect metabolic activity following liver cell metabolism, indicating that the assay can successfully differentiate between the active and inactive forms of prodrug compounds. In these results, the differential responses of the T47D and HepG2 cell lines is due to the basal metabolic differences imparted by the presence of cytochrome P450 enzymes between the two lines. Based on the biochemistry of the autobioluminescent reaction [6], it is unlikely that these differences would otherwise alter autobioluminescent output beyond manifesting as different basal signal output levels. However, these differences are controlled by normalizing the output of each cell line to its baseline autobioluminescent signal without compound treatment, which allows compound metabolic effects to be detected from different cell lines with minimal effects on assay sensitivity.

While these results show that the autobioluminescent phenotype is capable of functioning within this assay format, they do not indicate if the assay itself is reporting on a specific mechanism of action. Therefore, to determine if the metabolic impacts reported through autobioluminescent dynamics were specific or general in nature, the performance of this assay was correlated with ATP content, reactive oxygen species, and glutathione depletion assays. Autobioluminescence was found to correlate strongly with overall ATP content (Figure 2b), which has become the gold standard for tracking a cell’s overall metabolic activity level [23]. The similarity of these results and the improved detection capabilities of the autobioluminescent assay approach (Figure 2c) suggest that it is well suited to measuring general metabolic activity levels. 

Autobioluminescent dynamics were also observed to correlate well with the presence of reactive oxygen species (Figure 3), which if accumulated at high enough levels can alter intracellular signaling [24] and lead to the onset and progression of disease [25]. One exception was noted during this testing, where TBHP treatment of the HepG2 cell line was positively identified by the autobioluminescent assay, but not by the DCFH-DA assay. Given the otherwise strong agreement between these tests, and because this effect was only observed at the highest treatment level tested, it is likely that this detection discrepancy is the result of a difference in sensitivities between the two assays and not a fundamental difference in their detection abilities. However, in contrast to the assays that provide direct measurements of intracellular compounds, the autobioluminescent assay did not correlate with the secondary measurement marker glutathione. One possible explanation for these discrepancies between the autobioluminescent assay and the alterative assay formats is that the autobioluminescent luciferase enzyme relies on a larger number of co-factors than do the bioluminescent or fluorescent reporters used in the alterative assays. As such, the autobioluminescent assay can be triggered to report changes in metabolic activity resulting from alterations in intracellular O_2_, NADPH, ATP, or FMNH_2_ availability [26]. This suggests that the observed changes in autobioluminescence are not tied to a specific mechanism of action, but are rather the result of broad changes that directly affect the host cell’s basal metabolic rate. 

From a logistical perspective, the autobioluminescent assay foregoes the external substrate application requirements of the alternative assays, which obviates the need for sample destruction. The continuous output signal of the autobioluminescent cells allowed the samples to be prepared, placed into a plate reader, and monitored continuously for the full lifetime of the assay. This significantly reduced the hands-on time required for assay performance and required significantly fewer samples to be prepared to obtain the same number of data points. This increased the ease of use and decreased the reagent cost and hands-on time requirements of the assay, and also made this format more amenable to high throughput usage.

The results of the qRT-PCR tests show that the autobioluminescent response is not significantly influenced by the activation of the majority of phase I or phase II detoxification enzymes. Given that the autobioluminescent response correlates most strongly with primary metabolic activity markers, it is therefore likely that the onset of the autobioluminescent response occurs before sufficient time has elapsed to allow for upregulation of the phase II pathway enzymes under most treatment conditions. While these results demonstrate that the autobioluminescent data is therefore most indicative of CYP1A1 activation, they also suggest that there is a significant opportunity to develop biomarker-specific versions of the autobioluminescent system where the synthetic luciferase cassette is activated by the promoters of these alternative, currently non-responsive enzymes. This approach would provide a straightforward means for specifically tracking detoxification pathway activation in real time, which would be highly relevant for drug development applications.
